# The First Molecular Genotyping of *Naegleria fowleri* Causing Primary Amebic Meningoencephalitis in Thailand With Epidemiology and Clinical Case Reviews

**DOI:** 10.3389/fcimb.2022.931546

**Published:** 2022-07-13

**Authors:** Pannathat Soontrapa, Anupop Jitmuang, Pichet Ruenchit, Supathra Tiewcharoen, Patsharaporn T. Sarasombath, Chatchawan Rattanabannakit

**Affiliations:** ^1^ Division of Neurology, Department of Medicine, Faculty of Medicine Siriraj Hospital, Mahidol University, Bangkok, Thailand; ^2^ Division of Infectious Diseases and Tropical Medicine, Department of Medicine, Faculty of Medicine Siriraj Hospital, Mahidol University, Bangkok, Thailand; ^3^ Department of Parasitology, Faculty of Medicine Siriraj Hospital, Mahidol University, Bangkok, Thailand

**Keywords:** PAM, primary amebic meningoencephalitis, *Naegleria fowleri*, *Naegleria* spp., free-living ameba, genotyping, meningoencephalitis, meningitis

## Abstract

Primary amebic meningoencephalitis (PAM) is a rare and fatal central nervous system infection caused by *Naegleria fowleri*, a free-living amoeba found in the environment. To date, eight pathogenic *N. fowleri* genotypes have been reported worldwide. We aimed to explore the genotypes of *N. fowleri* that cause primary amebic meningoencephalitis in Thailand. In 2021, the 17th PAM case was reported, and a retrospective literature search of PAM cases in Thailand from 1982 through April 2021 was performed. Phylogenetic and genotyping analyses of the two mitochondrial (12S rRNA and 16S rRNA) and nuclear (ITS1 and 5.8s rRNA) genes of *N. fowleri* were performed on four available clinical isolates. Based on the mitochondrial and nuclear genes, *N. fowleri* genotype T3 was found to cause PAM in three out of four cases. However, disagreement between the genotype based on the mitochondrial and nuclear genes was found in one of the PAM cases, in which the 12S rRNA locus suggested the causative genotype as T1, while the ITS1 implied genotype T4. The discrepancy between the mitochondrial and nuclear genome was previously observed, which suggests the possible horizontal gene transfer among *N. fowleri* species. Based on the ITS1 gene, two *N. fowleri* genotypes, T3 and T4, were found to be the genotypes causing PAM in this study. In addition, *N. fowleri* genotype T2 was previously reported in a traveler who was infected in Thailand. Thus, at least three genotypes (T2, T3, and T4) of *N. fowleri* are found to be associated with PAM in Thailand.

## Introduction

Primary amebic meningoencephalitis (PAM) is a rare fatal central nervous system infection caused by *Naegleria fowleri*, a free-living ameba found in the environment mainly in soil and freshwater (Visvesvara, 2014). *N. fowleri* mainly enters the human body *via* nasal aspiration of the trophozoites (Visvesvara, 2014). It invades the central nervous system through the cribriform plate and olfactory nerves resulting in cerebral necrosis and edema (Visvesvara, 2014). A presumptive diagnosis of PAM is made directly by observing ameba with blunt-end semi-spherical pseudopodia during microscopic examination of cerebrospinal fluid (CSF) or brain tissue (Cope and Ali, 2016). A definite diagnosis of the disease is based on immunohistochemistry (IHC), indirect immunofluorescence assay (IFA), or molecular diagnosis from clinical specimens (Cope and Ali, 2016). From 1962 to 2018, a total of 381 PAM cases were reported and 182 cases were confirmed and fit the diagnosis criteria for definite PAM (Gharpure et al., 2021). *Naegleria fowleri* has been identified in only two countries in Southeast Asia, including Thailand and Vietnam (Gharpure et al., 2021). Since a warm environment favors the growth of *N. fowleri*, the majority of PAM cases are found in tropical regions. However, most PAM cases have been reported in subtropical and temperate regions. This suggests that the disease is likely to be underestimated or underreported in tropical regions due to the lack of trained laboratory personnel and equipment. Recently, molecular diagnostic techniques have become available in several laboratories in developing countries, allowing researchers in different parts of the world to explore the differences in *N. fowleri* genotypes and their association with disease phenotypes and geographic distribution. Several genotyping systems based on different gene targets including 12S ribosomal RNA (12S rRNA), 16S ribosomal RNA (16S rRNA), internal transcribed spacers (ITS1), and 5.8S ribosomal RNA (5.8S rRNA) have been proposed to classify *N. fowleri* (De Jonckheere, 1998; Pélandakis et al., 2000; Tiewcharoen et al., 2007; De Jonckheere, 2011). However, the most well-known genotyping system for *N. fowleri* based on the length of ITS1 and a base transition in the 5.8S rDNA sequences has been proposed by several laboratories which categorize *N. fowleri* into eight genotypes (T1-T8) (De Jonckheere, 2011). It has been hypothesized that distinct genotypes may have different geographic distribution patterns or associate with pathogenicity (Zhou et al., 2003). Different geographic distribution of each genotype isolated from the environments and clinical specimens has been reported (Pélandakis et al., 2000; De Jonckheere, 2011). In Europe, up to seven genotypes (T2-T8) have been detected (De Jonckheere, 2011). Three genotypes (T1-T3) and two genotypes (T2-T3) were previously reported in the USA and mainland Asia (except Japan), respectively; while only one genotype (T5) was found in Oceania and Japan (De Jonckheere, 2011). Until now, *N. fowleri* genotype T3 seems to be the most abundant strain (De Jonckheere, 2011). No association between the *N. fowleri* genotype and virulence has been observed so far (De Jonckheere, 2011). However, additional data is needed to conclude whether there is an association between the genotype and the degree of virulence.

Fifteen cases of PAM have been reported in Thailand, and one case had strong evidence associated with the acquisition of infection in Thailand. In 2021, the 17th fatal PAM case was reported in Thailand. Genotyping analysis of the causative isolates of PAM in Thailand has not been performed previously. We performed molecular characterization and phylogenetic analysis of the genotypes of cases reported in Thailand and compared them with other available data of clinical and environmental isolates on the Genbank database. In addition, the clinical and laboratory findings of case No.17 are described. All available data on *N. fowleri* infection cases in Thailand were thoroughly reviewed.

## Materials and Methods

### Samples and Patient Information

A retrospective literature review and chart review of reported cases of *N. fowleri* infection (Cases No.1-16) in Thailand from 1982 to April 2021 was performed (Jariya et al., 1983; Somboonyosdej and Pinkaew, 1987; Charoenlarp et al., 1988; Sirinavin et al., 1989; Poungvarin and Jariya, 1991; Chotmongkol et al., 1993; Wattanaweeradej et al., 1996; Petchsuwan and Ruengsuwan, 1997; Viriyavejakul et al., 1997; Bunjongpuk, 2000; Sithinamsuwan et al., 2001; Siripanth et al., 2005; Pudthasa et al., 2016; Stubhaug et al., 2016). In addition, the recent PAM case No.17 has been thoroughly reviewed and presented. This study was conducted in the Faculty of Medicine Siriraj Hospital, Mahidol University, Bangkok, Thailand. The study protocol was approved by the Siriraj Institutional Review Board of Research involving Human Subjects (SIRB) (COA no. Si 492/2021). Informed consent was obtained from the patient’s husband before writing the report. Molecular characterization and phylogenetic analyses of all available *N. fowleri* strains previously reported and the strain from the current PAM case (No.17) was performed and compared with those available in the National Center for Biotechnology Information (NCBI) database.

### DNA Isolation

DNA samples of previously reported Thai cases (cases No.2, SI1 isolate; No.5, RA isolate; and No.8, CC isolate) were obtained from axenic culture stocks kept in liquid nitrogen. The stock isolates were thawed and maintained in axenic Nelson’s medium until they reached approximately 80% confluence. The amoeba isolates were washed and pelleted for DNA extraction. DNA extraction of *N. fowleri* strain from patient No.17 (SI2 isolate) was performed directly from the patient’s CSF sample. Extraction was performed using the QIAmp DNA Mini Kit (Qiagen, Hilden, Germany), following the manufacturer’s protocol. DNA purity and concentration of each sample were measured using a NanoDrop 2000 UV-Vis Spectrophotometer (Thermo Fisher Scientific, Waltham, MA, USA). DNA samples were subjected to PCR and DNA sequencing.

### PCR Amplification and DNA Sequencing

PCR targeting two mitochondrial genes (12S rRNA small subunit and 16S rRNA large subunit), and one genomic locus (the partial 18S rRNA, ITS1, 5.8s rRNA, ITS2) of *Naegleria fowleri* were amplified using specific primers and PCR cycling conditions, as listed in **Supplementary Table S1**. PCR was performed using the AccuStart II PCR SuperMix kit (Quantabio, Beverly, MA, USA). The reactions were set up in a volume of 20 µL, containing 2 µL of 2x AccuStrat PCR buffer, 0.4 µL of each primer, 10 ng of DNA, and H_2_O up to 20 µL. The PCR cycling conditions are listed in **Supplementary Table S1**. The PCR amplicons were further analyzed by DNA sequencing.

### Phylogenetic and Genotyping Analyses

Nucleotide sequences of 12S rRNA, 16S rRNA, and ITS were blasted, aligned, and compared with reference genotypes deposited in the GenBank database using the BLAST 2.0 program (National Center for Biotechnology Information (NCBI), Bethesda, MD, USA). Nrooted phylogenetic trees were derived using the maximum likelihood ratio. Evolutionary analyses were conducted using Molecular Evolutionary Genetics Analysis (MEGA) software version 11.0 (Tamura et al., 2021).

## Results

### Clinical Manifestations of the Current Case No.17

**Figure 1:**
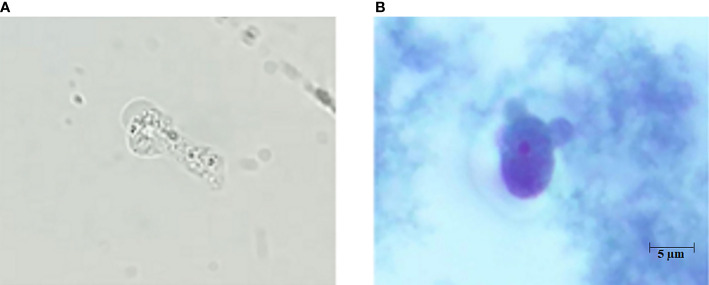
**(A)** Direct CSF examination of case No.17 showed *N. fowleri* trophozoite with hyaline semi-spherical pseudopods, approximately 10-20 µm in diameter. Its cytoplasm contained many granules and vacuoles. (Courtesy of Dr. Thamrongkiat Amornvivattanakul, Aikchol Hospital) **(B)** Gomori’s trichrome staining of the ameba trophozoite found in the patient’s CSF. *N. fowleri* trophozoite with a single nucleus containing a large, central karyosome surrounded by halo.

**Figure 2:**
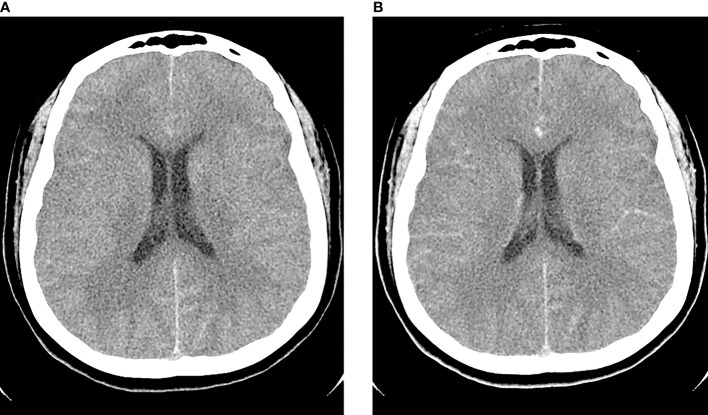
Axial pre-contrast **(A)** and post-contrast **(B)** computed tomography (CT) of the brain on day 3 post-infection showed generalized edema with post-contrast leptomeningeal enhancement.

A 40-year-old woman presented with an acute onset of severe headache radiating to the occiput and high-grade fever for 24 hours. She had underlying allergic rhinitis and recurrent sinusitis that required regular nasal irrigation with sterile saline. Three days before the onset of symptoms, she had been to the Krok E-Dok waterfall in Saraburi province, where her family usually went on vacation every weekend. Because of her belief that natural water is sacred, like holy water, she poured water from this waterfall on her head and face. She was admitted to the hospital and initially diagnosed with acute febrile illness of unknown origin. Empirical antibiotics, ceftriaxone and doxycycline, were given. Two days after admission, she developed a neck stiffness, nausea, and vomiting. A lumbar puncture was performed, which showed clear, colorless cerebrospinal fluid (CSF) with elevated opening pressure. Her CSF revealed pleocytosis with a white blood cell count of 653/mm^3^ (neutrophils 11% and lymphocytes 89%), a red blood cell count of 688/mm^3^, a decreased glucose level (CSF glucose 63 mg/dL, CSF to blood glucose ratio of 0.37), an increased protein level (247 mg/dL), and numerous fast-moving amebic-like organisms with active semi-spherical pseudopodia were found by direct CSF examination (**Figure 1A**, and **Supplementary Video S1**). Trichrome staining of the organism resembled an ameba-like organism with semi-spherical pseudopodia. It had a single nucleus that contained a large central karyosome surrounded by a clear halo, similar to that of *Naegleria* spp. (**Figure 1B**). Identification of the causative species by PCR was positive for *N. fowleri*, but negative for *Acanthamoeba* spp. and *Balamuthia mandrillaris*. The CSF culture for bacteria was negative. Due to the high suspicion of primary amebic meningoencephalitis, the patient was administered intravenous amphotericin B, dexamethasone, rifampicin, fluconazole, and azithromycin. The patient was transferred to our tertiary care hospital for further intensive care. Brain computed tomography (CT) on the first day showed unremarkable findings; however, on the third day after the onset of symptoms, generalized brain edema with uncal herniation, generalized leptomeningeal enhancement, and chronic right maxillary sinusitis were observed (**Figure 2**). An organism compatible with *Naegleria* sp. was grown on non-nutrient agar with *Escherichia coli* (NNE) on day three after inoculation. Miltefosine was prescribed for compassionate use in this case on the fourth day of illness, courtesy of the National Vector Borne Disease Prevention and Control Program, Ministry of Public Health of Thailand. Unfortunately, her condition deteriorated over the following 12 hours to a comatose status with concomitant seizures. The patient died 5 days after hospital admission due to brain herniation. Her husband and family refused an autopsy.

### PAM Cases Previously Reported in Thailand

Between 1983 and December 2021, 17 cases of *N. fowleri* infection occurred in Thailand: 15 previously reported cases, one unpublished case, and one current case. The demographic data, clinical characteristics, and laboratory findings of the patients are presented in **Table 1**. The clinical data for Case no.8 were missing. Thus, this case was not included in the analysis of the clinical data. Fifteen patients presented with primary amoebic meningoencephalitis, with the exception of one patient (No.13) who had only nasal infection without evidence of neurological involvement. Ten out of 16 (62.5%) patients were male with a median age of 20.3 ± 20.9 years, ranging from 8 months to 71 years. Eleven of 16 (68.8%) were children and young adults under 19 years of age. Most cases were previously healthy, except one with obesity, one with chronic sinusitis, and one with a history of recurrent sinusitis. Most infections occurred in the central region (43.8%), followed by the eastern (25%) and northeastern (31.3%) regions of Thailand. Two cases (No.8 and No.13) did not have data on the infection sites. No cases were reported in the northern, southern, or western regions of Thailand. A history of water activity, such as swimming in freshwater or using nonsterile water for nose irrigation, was primarily associated with the infection in most cases. The current case (No.17) had a history of water splashing and pouring water only on her head without evidence of nasal aspiration. Five patients (No.4, No.6, No.7, No.12, and No.13) had no history of water exposure. Ten cases (62.5%) were infected in the summer, two (12.5%) in the rainy season, and three (18.8%) in the winter. Thirteen cases were diagnosed based on the observation of the organism in the CSF by microscopic-based methods; three cases (No.11, No.12, and No.16) were diagnosed postmortem, and one case (No.16) was diagnosed based on the molecular diagnosis of the patient’s CSF. Most CSF profiles of the patients revealed elevated white blood cells (median 900 cells/mm^3^, ranging from 226 to 23,000), with neutrophil predominance (median 90%, ranging from 0-100%), increased red blood cells (median 744 cells/mm^3^, ranging from 100 to 4850), elevated protein levels (median 337.5 mg/dL, ranging from 53 to 575), and decreased glucose levels (median 20 mg/dL, ranging from 0 to 204). Only three cases (No.6, No.7, and No.13) (18.8%) were completely cured including one case (No.14) without neurological involvement (**Table 1**). Two cases (No.6 and No.7) were cured with a combination of intravenous amphotericin B, oral rifampicin, and other azole-type antifungal drugs.

**Table 1:** Demographic data, laboratory findings and clinical characteristics of reported *N. fowleri* infection cases in Thailand.

No.	Month and year	Sex	Age (years)	U/D	District/ Province/ Region	Source/ Exposure	Season	Onset	Initial CSF profile	Medication	Outcome	Diagnostic methods	Molecular identification of the causative strain	Reference No.
1	September, 1982	M	5	No	Kanthararom/ Sisaket/ North eastern	Pond swimming	Rainy	4 d	WBC 226 cells/mm^3^ (PMN 94%), RBC 800 cells/mm^3^, P 53 mg/dL, G 2.5 mg/dL,	No	dead	DE	N/A	Jariya et al., 1983
2	May, 1986	M	17	No	Rat Burana/ Bangkok/ Central	Water splashing	Summer	3 d	WBC 13,155 cells/mm^3^ (PMN 82%), RBC 4,850 cells/mm^3^, P 432 mg/dL, G 22 mg/dL	IV & IT Amp B, Oral Rifampicin, Sulfadiazine, Tetracycline	dead	DE, NNE, brain histology	Performed in this study	Charoenlarp et al., 1988
3	March, 1986	M	14	No	Muang/ Trat/ Eastern	Pond swimming	Summer	2 d	WBC 540 cells/mm^3^, P 610 mg/dL, G 0 mg/dL	IV penicillin, Oral Metronidazole, chloramphenicol	dead	DE	N/A	Somboonyosdej and Pinkaew, 1987
4	April, 1986	M	8 mo	No	Bo Rai/ Trat/ Eastern	No	Summer	3 d	WBC 2,000 cells/mm^3^ (PMN 100%), P 360 mg/dL, G 20 mg/dL	IV Gentamicin, Oral Rifampicin, Ampicillin, Metronidazole	dead	DE	N/A	Somboonyosdej and Pinkaew, 1987
5	May, 1987	F	4.5	No	Nakhon Pathom/ Central	Canal swimming	Summer	3 d	WBC 450 cells/mm^3^ (PMN 91%), RBC 480 cells/ml, P 715 mg/dL, G 2 mg/dL	IV Amp B, Oral Rifampicin	dead	DE, NNE	Performed in this study	Sirinavin et al., 1989
6	1991	M	61	No	Srisaket/ North eastern	No	Summer	4 d	WBC 700 cells/mm^3^ (PMN 84%), RBC 1,960 cells/mm^3^, P 102 mg/dL, G normal	IV Amp B, Oral Rifampicin, Ketoconazole	cure	DE	N/A	Poungvarin and Jariya., 1991
7	May, 1992	F	18	No	Ubonratchathani/ North eastern	No	Summer	5 d	WBC 900 cells/mm^3^ (PMN 90%), P 106 mg/dL, G 42 mg/dL	IV Amp B, Oral Rifampicin, Itraconazole	cure	DE, Page’s saline culture	N/A	Chotmongkol et al., 1993
8	Unpublished	N/A	N/A	N/A	Chachoengsao/ Eastern	N/A	N/A	N/A	N/A	N/A	dead	DE, NNE	Performed in this study	This study
9	1996	M	5	N/A	N/A/ Central	N/A	Summer	N/A	N/A	IV Amp B, Oral Rifampicin	dead	DE	N/A	Wattanaweeradej et al., 1996
10	1997	M	12	No	Samut Prakan/ Central	Canal Swimming	Summer	2 d	WBC 23,000 cells/mm^3^ (PMN 95%), RBC 100 cells/mm^3^, P 588 mg/dL, G 146 mg/dL	none	dead	DE	N/A	Viriyavejakul et al., 1997
11	1997	F	9	N/A	Nakorn Pathom/ Central	Canal swimming in Nakorn Pathom and Petchaburi	Summer	N/A	WBC 900 cells/mm^3^, P 315 mg/dL, G 204 mg/dL	none	dead	Autopsy (Postmortem)	N/A	Petchsuwan and Ruengsuwan, 1997
12	2000	F	9	N/A	N/A/ Northeastern	No	Summer	N/A	N/A	none	dead	Autopsy (Postmortem)	N/A	Bunjongpuk et al., 2000
13	February, 2001	M	25	No	Bangkok/Central	No	Winter	4 d	WBC 1,500 cells/mm^3^ (PMN 79%), P 69 mg/dL, G 1 mg/dL	IV & IT Amp B, Rifampicin	dead	DE	N/A	Sithinamsuwan et al., 2001
14*	2005	M	N/A	Chronic sinusitis	N/A	Pond swimming	N/A	2-3 d	NA	N/A	cure	DE, Giemsa stain of nasal exudate	N/A	Siripanth et al., 2005
15	2014	M	12	Obesity	Udon Thani/ North eastern	Pool swimming	Rainy	1 wk	WBC 7,040 cells/mm^3^,P 159 mg/dL, G 5 mg/dL	none	dead	CSF staining	Genotype T2 (KT375442)	Pudthasa et al., 2016
16**	2016	F	71	N/A	Chonburi or Trat/ Eastern	Nasal irrigation using tap water	Winter	12 d	WBC 2,115 cells/mm^3^ (PMN 87%), P 690 mg/dL, G 200 mg/dL	none	dead	Autopsy (Postmortem)	N/A	Stubhaug et al., 2016
17	January 2021	F	40	Recurrent sinusitis	Saraburi/ Central	Waterfall splashing	Winter	3 d	WBC 653 cells/mm^3^ (L 91%), RBC 688 cells/mm^3^,P 247 mg/dL , G 63 mg/dL	IV Amp B, Dexamethasone, Oral rifampicin, Fluconazole, Azithromycin	dead	DE, Trichrome stain, NNE, PCR	Performed in this study	This study

*Case No. 14 has only local N. fowleri infection without neurological involvement. **case No.16: Norwegian patient infected in Thailand. Abbreviation: M, male; F, female, N/A, not available; d, day; wk, week; mo, month; WBC, white blood cells; RBC, red blood cells; PMN, polymorphonuclear cells; L, lymphocyte; P, protein; G, glucose; IV, intravenous; IT, intrathecal; Amp, Amphotericin B; NNE, non-nutrient agar with Escherichia coli culture; PCR, polymerase chain reaction; DE, direct CSF examination.

### Subgroup Analysis Based on 12S rRNA and 16S rRNA Loci Among *N. fowleri* Species Causing PAM in Thailand

**Figure 3:**
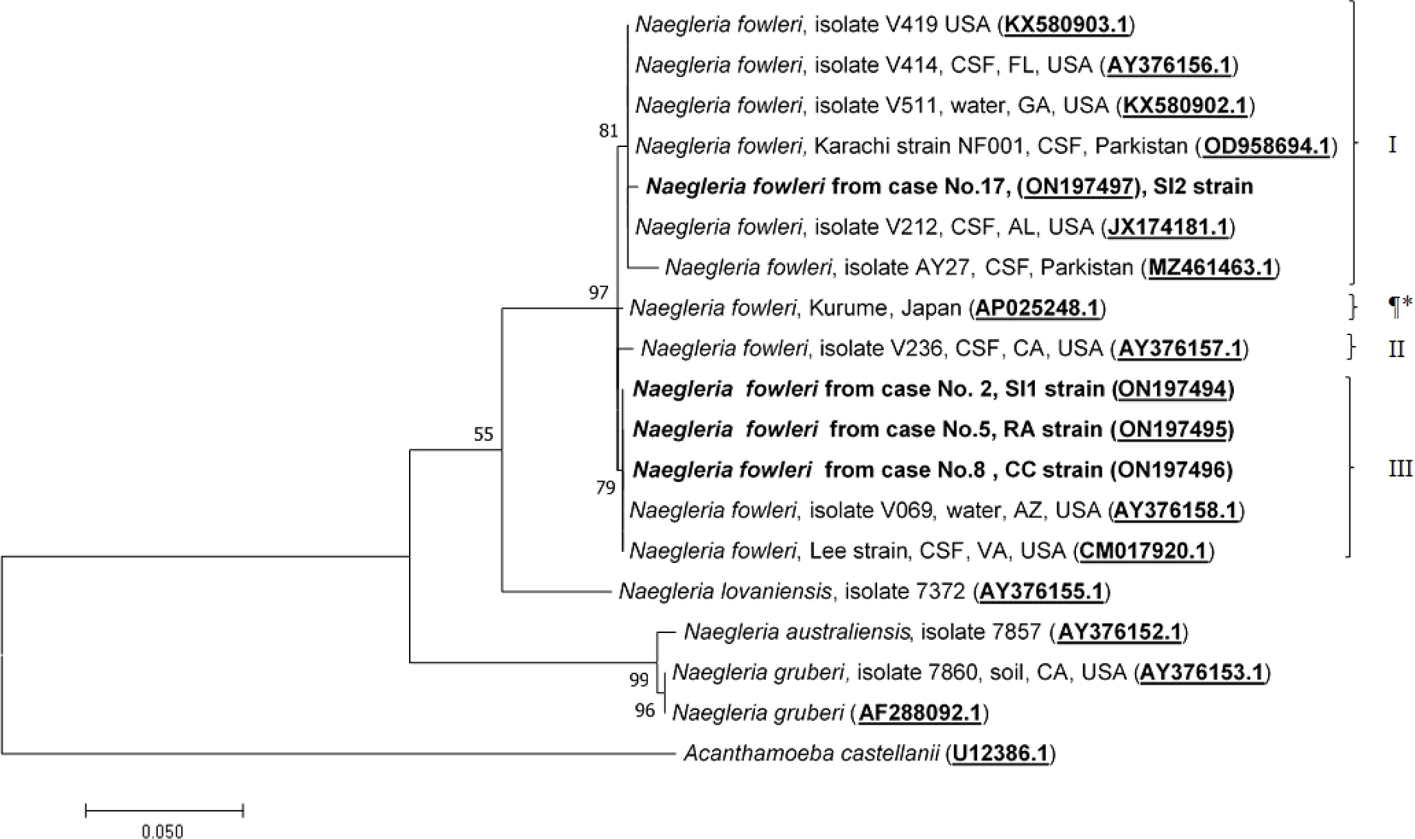
Phylogenetic relationships on the 12S rRNA sequences among the *N. fowleri* isolates. *N. fowleri* subgroups based on Zhou et al^8^ are listed next to the branches. The subgroup with * was not previously classified by this method. The tree was constructed using the maximum-likelihood method based on the Tamura-Nei model. ^23^ There were a total of 614 nucleotides in the final dataset.

**Figure 4:**
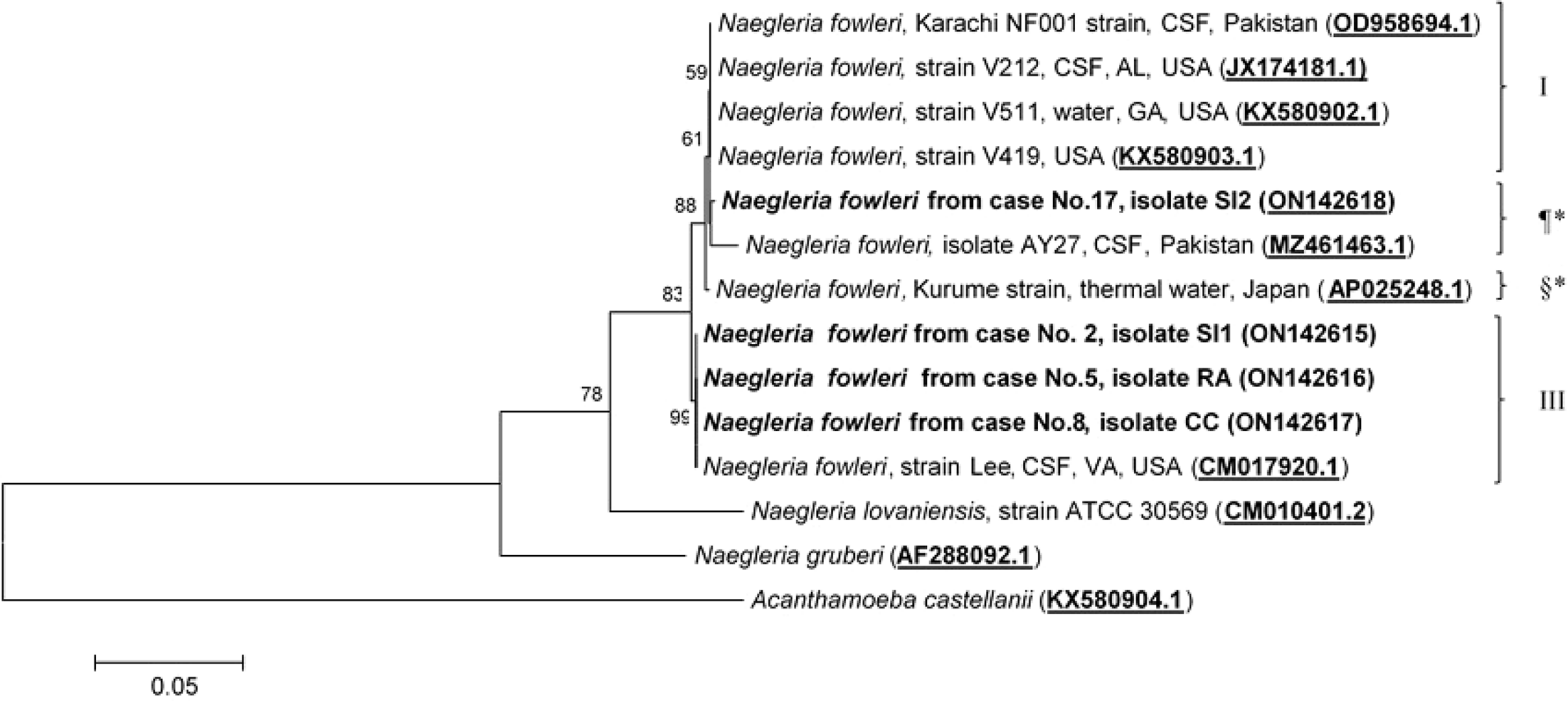
Phylogenetic relationships on the 16S rRNA sequences among the *N. fowleri* isolates. *N. fowleri* subgroups based on Zhou et al ^8^ are listed next to the branches. The subgroups with * were not previously classified by this method. The tree was constructed using the maximum-likelihood method based on the Tamura-Nei model.^23^ There were a total of 772 positions in the final dataset.

Molecular characterization and phylogenetic analyses of the isolates (Cases No.2, SI1 isolate; No.5, RA isolate; No.8, CC isolate; and No.17 SI2 isolate) causing PAM in Thailand were performed on the two mitochondrial genes (12S rRNA and 16S rRNA) of *N. fowleri.* The nucleotide sequences of the 12S rRNA of cases No.2, No.5, and No.8 were 99.8%, 99.8%, and 100% identical to *N. fowleri* isolate V212 (AY376157.1), respectively, whereas in case No.17, the sequence was 99.5% homologous with *N. fowleri* strain V419 (KX580903.1) (**Figure 3**). The 16S rRNA sequences of cases No.2, No.5, and No.8 were 100%, 100%, and 99.5% identical to *N. fowleri* isolate Lee (CM017920.1), whereas the 16S rRNA of the isolate from case 17 was 99% compatible with *N.fowleri* isolate AY27 (MZ461463) (**Figure 4**). The sequences of cases No.2, No.5, No.8, and No.17 were submitted to NCBI under accession numbers 12S rRNA: ON197494, ON197495, ON197496, ON197497, and 16S rRNA: ON142615, ON142616, ON142617, and ON142618, respectively.

Four major subgroups were categorized based on the 12S rRNA and 16S rRNA loci (**Figures 3**, **4**). Based on the sequences of the two mitochondrial genes, three *N. fowleri* isolates from cases No.2, No.5, and No.8 clustered together in one lineage, previously described as genotype 3 (T3) by Zhou et al. (Zhou et al., 2003). However, the isolate from case No.17 was categorized in another subgroup, closely related to *N. fowleri* isolate AY27 (MZ461463) from the CSF of a patient in Pakistan.

### Genotyping and Phylogenetic Analysis Based on ITS1 and 5.8S rRNA Among *N. fowleri* Species Causing PAM in Thailand

**Figure 5:**
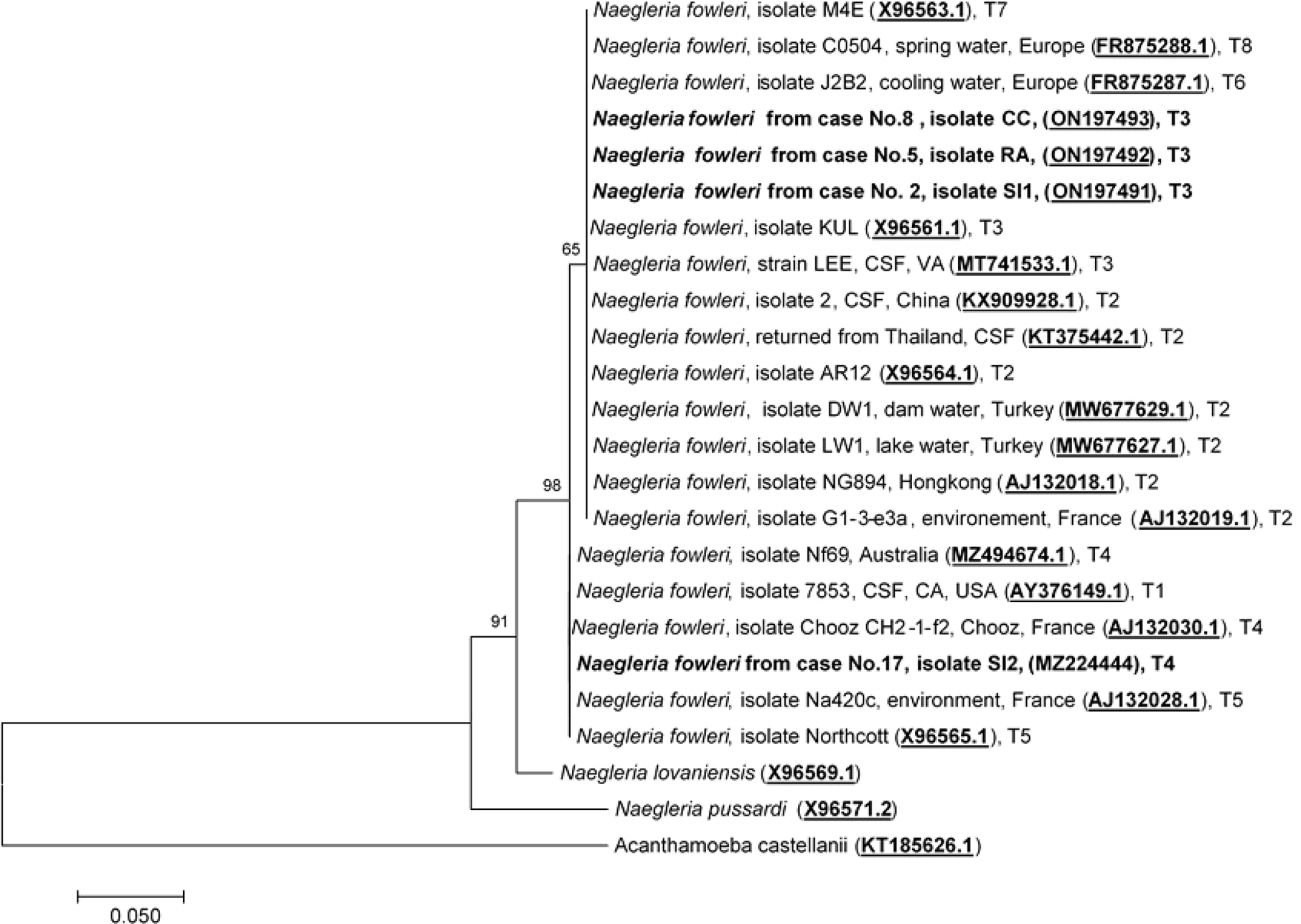
Phylogenetic relationships on the ITS1 and 5.8S rRNA sequences among the *N. fowleri* isolates. The tree was constructed using the maximum-likelihood method based on the Tamura-Nei model.^23^ There were a total of 124 positions in the final dataset.

To date, eight pathogenic *N. fowleri* genotypes, T1 to T8, reported worldwide, have been categorized based on the length of the ITS1 and the base at position 31 in the 5.8S rRNA sequences (De Jonckheere, 1998; Pélandakis et al., 2000; Zhou et al., 2003; De Jonckheere, 2011). Analysis of the ITS1 sequences of cases No.2, No.5, and No.8 was previously performed by Tiewcharoen et al. (Tiewcharoen et al., 2007) but had not been submitted to NCBI. These isolates were reperformed in this study and submitted to NCBI. The ITS1 sequences of cases No.2, No.5, and No.8 were 99.6%, 99.3%, and 99.1% identical, to *N. fowleri* isolate Lee (MT741533.1), respectively. The ITS1 sequences of these cases were compatible with *N. fowleri* genotype T3, which has an ITS1 sequence length of 86 bp and a T nucleotide at position 31 in 5.8S rRNA. The ITS1 nucleotide sequence of the current case No.17 was 100% identical to *N. fowleri* isolate Nf 69 (MZ494674.1). The sequence length of the ITS1 gene in case No.17 was 86 bp, with the C nucleotide at position 31 in the 5.8S rRNA, which indicates genotype T4 as the causative genotype. The ITS1 sequences of cases No.2, No.5, No.8, and No.17 were submitted to NCBI under the GenBank accession numbers ON197491, ON197492, ON197493, and MZ224444, respectively. Two genotypes, T3 and T4, were found to cause PAM in Thailand, with genotype T3 (Cases No.2, No.5, and No.8) being the most prominent phenotype found in this study, and genotype T4 was the causative genotype of the current case (No.17). *N. fowleri* genotype T4 has never been reported in Thailand and was first reported in this study. Because the length of ITS1 sequences varied depending on *N. fowleri* genotypes, phylogenetic analysis of ITS1 and 5.8S rRNA was performed based on the consensus 127 nucleotide sequence of the partial part of ITS1 and 5.8S rRNA. Phylogenetic analysis of ITS1 and 5.8S rRNA categorized *N. fowleri* genotypes into two main clades: Clade 1: T1, T4, and T5, and Clade 2: T2, T3, T6, T7, and T8, as shown in **Figure 5**.

## Discussion

*Naegleria fowleri* is a free-living thermophilic ameba commonly found in the environment such as soil and natural freshwater sources (Visvesvara, 2014). It has been found on every continent of the world except Antarctica (De Jonckheere, 2011). It is a causative agent of a fatal disease called primary amebic meningoencephalitis (PAM) (Visvesvara, 2014). Transmission occurs through the nasal passages during swimming, water activity, or nasal irrigation with contaminated water, in which ameba migrates to the cribriform plate *via* the olfactory nerves to the central nervous system and rapidly causes extensive damage to the brain parenchyma and meninges (Visvesvara, 2014). The majority of PAM cases worldwide are children and young adults, consistent with the data found in this study (Cope and Ali, 2016). Besides having a water exposure history, a couple cases (No.14 and No.17) also had chronic or recurrent sinusitis, which may make them more susceptible to the infection. Interestingly, *N. fowleri* trophozoites were found in the nasal exudate of one patient (No.14) without neurological involvement. Thus, amoeba colonization may occur in individuals with chronic or recurrent sinusitis. It is not known if this patient would be more susceptible to developing PAM. The exposure history of infection in our current case (No.17) was unclear because she used only sterile nasal saline for nose irrigation, in addition to pouring waterfall water on her head.

Thailand is in a subtropical climate zone. The temperature in the northern region of Thailand is slightly lower than that in the central and southern regions. In Thailand, the highest number of cases occurred during the summer, which is consistent with data from other regions of the world. Increased exposure during water activities in the summer may increase the risk of infection (Visvesvara, 2014; Cope and Ali, 2016). Moreover, the ambient temperature may affect the ability of amoeba transmission (Cope and Ali, 2016). It has been proposed that global warming may affect the number or pattern of disease transmission (Cope and Ali, 2016). In the US, in the past decade, PAM cases outside of the southern-tier states have been increasing (Cope and Ali, 2016). Neurological manifestations of PAM are the result of fulminant inflammation of the brain parenchyma and meninges caused by the immune response to the amoeba, which includes headache, fever, alteration of consciousness, signs of increased intracranial pressure, and signs of meningitis. Typical CSF findings in PAM cases are similar to those of bacterial meningitis, including increased white blood cells with neutrophil predominance, increased red blood cells, elevated protein, and decreased glucose (hypoglycorrhachia) levels, except the presence of numerous fast-moving ameba which is usually observed during direct CSF examination. Why prominent CSF lymphocytes are observed in some individuals remains unclear. Although numerous ameba can be found in the cerebrospinal fluid of infected individuals, the diagnosis of this disease is challenging because ameba trophozoites may be overlooked or dismissed as leukocytes. In approximately 50% of the cases in this study, the diagnosis of PAM was delayed, the patients were treated as having bacterial meningitis, and all of these patients died within 1 week. Due to the lack of advanced laboratories in some developing countries, molecular identification of ameba from patients’ CSF may not be available. Thus, the diagnosis is usually delayed or misdiagnosed.

Pathogenic *N. fowleri* has been reported in the central, northern, and southern regions of Thailand in several provinces, including Bangkok, Chumphon, Nakhon Nayok, Nakhon Sawan, Prachuap Khiri Khan, Saraburi, Sukhothai, Surat Thani, and Ratchaburi (Tiewcharoen et al., 2007; Junnu et al., 2018). Most cases were from central Thailand, followed by northeastern and eastern Thailand. It is possible that in other regions of Thailand, *N.fowleri* or PAM cases may exist but are not recognized. The mainstay of PAM treatment includes early diagnosis, combination treatment, and proper management of elevated intracranial pressure. Empirical antibiotic treatments have been used in most reported cases. To date, only seven confirmed PAM survivors have been reported worldwide (Cope and Ali, 2016; Gharpure et al., 2021). Intravenous and intrathecal amphotericin B, azoles, rifampin, azithromycin, miltefosine, and dexamethasone were used in patients that survived (Gharpure et al., 2021). A few survivors also received other treatments including induced hypothermia and decreased intracranial pressure by extraventricular drainage, hyperosmolar therapy with mannitol, and 3% saline (Cope and Ali, 2016). Because miltefosine is not listed on the Thai Food and Drug (FDA) approved medicine, it was prescribed for compassionate use in this patient. Thus, most PAM cases in Thailand did not receive this medication. While the current case (No.17) received combination treatment on day 2 and miltefosine on day 4 post-onset, the patient still died from brain herniation, which emphasizes the need for early diagnosis and treatment.

The causative genotypes of *N. fowleri* were identified in this study by molecular characterization of two mitochondrial genes (12S rRNA and 16S rRNA) and one nuclear locus (ITS1 and 5.8S rRNA). It has been shown previously that mitochondrial genes can be used for *N. fowleri* genotyping. Based on the phylogenetic analysis of the 12S rRNA and 16S rRNA sequences, *N. fowleri* was classified into four subgroups. Three of the cases (cases No.2, No.5, and No.8) were classified as genotype T3, and case No.3 was classified as genotype T1, based on the classification by Zhou et al. (Zhou et al., 2003). Until now, eight pathogenic *N. fowleri* genotypes (T1 to T8) have been reported worldwide based on the length of ITS1 and the base at position 31 in the 5.8S rRNA sequences (Pélandakis et al., 2000; De Jonckheere, 2011). The difference in the length of ITS1 is due to the number of short repeats (Pélandakis et al., 2000; De Jonckheere, 2011). The nucleotide sequence of the 5.8S rRNA among *N. fowleri* species is truly conserved, with a length of 175 bp, except for the variation of either C or T at the 31 nucleotide position. It is important to note that all genotypes had the same ITS2 sequence (106 nucleotides). Based on the 12S rRNA and ITS1 genotyping, *N. fowleri* genotype 3 was found to be the causative species in cases No.2, No.5, and No.8. However, the 12S rRNA and ITS1 sequences showed disagreement in the genotype between each other (**Figures 3**, **5**). Interestingly, this discrepancy was also observed in the isolate V1005 from the PAM patient in Australia on both mitochondrial 12S rRNA and nuclear ITS1 genes (Joseph et al., 2021). It was previously reported that genotyping based on 12S rRNA and ITS1 was consistent between the two loci (Zhou et al., 2003). However, in this study, we found inconsistent genotyping results for case No.17 between the two loci. The discrepancy of the 12S rRNA and ITS1 in *N. fowleri* genotyping was also observed in the other study based on the whole mitochondria and nuclear genome analysis, which suggested that horizontal gene transfer in the mitochondrial genome may occur within the species (Joseph et al., 2021). It is also possible that most reported isolates were identified based on the ITS1 sequences, and highly diverse genetic variations were observed in the ITS1 and 5.8S rRNA regions. Based on the ITS1 sequence, *N. fowleri* genotype 4 was the cause of PAM in Case No.17. In addition to genotypes T3 and T4 found in this study, *N. fowleri* genotype T2 was reported to be the causative agent in the Norwegian traveler case (No.16) who had a history of water exposure before the onset of symptoms that supported that the infection occurred during a trip in Thailand (Stubhaug et al., 2016). Thus, at least three genotypes (T2, T3, and T4) of *N. fowleri* were found to be associated with PAM cases in Thailand. In this study, *N. fowleri* genotype T4 was identified as the causative agent of PAM for the first time in Thailand. *N. fowleri* genotypes T1, T2, T3, and T5 were most commonly found in PAM cases (De Jonckheere, 2011; Joseph et al., 2021). This is likely due to their prevalence in the environment. A few surveys of the presence of *N. fowleri* in the environment in Thailand were previously reported (Tiewcharoen and Junnu, 2001; Tiewcharoen et al., 2007; Junnu et al., 2018). It was found in freshwater sources in several provinces of Thailand including Bangkok, Chumphon, Ratchaburi, Nakhon Nayok, Nakhon Sawan, Prachuap Khiri Khan, Saraburi, Sukhothai, and Surat Thani (Tiewcharoen and Junnu, 2001; Tiewcharoen et al., 2007; Junnu et al., 2018). However, only one survey that the genotype of the environmental isolate was performed in which only genotype T3 was found (Tiewcharoen et al., 2007). *N. fowleri* genotype T1 was reported to cause PAM in patients in the US (Zhou et al., 2003) and a US patient returning from India (Harris et al., 2021). Other genotypes, including T6, T7, and T8, were only isolated from the environments in Europe, but not from clinical samples (De Jonckheere, 2011). However, this study has some limitations. Only four out of 17 N*. fowleri* isolates could be retrieved and no environmental isolates were included for genotyping in this study. Thus, it is difficult to determine the prevalence or the association of different genotypes and geographic distribution in Thailand. Further genotyping studies of clinical and environmental isolates are required to determine such association. Genotype identification based on ITS1 locus could help indicate the source of patient infection and understand the epidemiology of disease transmission. Due to the limited number of studies, there is insufficient evidence to conclude that there are differences in the phenotypes or pathogenicity of each genotype. In addition, the reasons why some genotypes were found only in the environments is still needed to be explored. Further studies are required to determine the differences in the virulence of each genotype. To the best of our knowledge, this is the first genotyping study of *N. fowleri* clinical isolates with detailed epidemiological, clinical, and molecular reviews of PAM cases in Thailand.

## Data Availability Statement

The datasets presented in this study can be found in online repositories. The name of the repository and accession numbers can be found below: Genbank, NCBI; ON197494-197497, ON142615-142618, ON197491-197493, and MZ224444.

## Ethics Statement

The studies involving human participants were reviewed and approved by the Siriraj Institutional Review Board of Research involving Human Subjects (SIRB) (COA no. Si 492/2021). Written informed consent for participation was not required for this study in accordance with the national legislation and the institutional requirements. Written informed consent was obtained from the individual(s) for the publication of any potentially identifiable images or data included in this article.

## Author Contributions

PS, AJ, and CR performed the clinical data review. PR, ST, and PTS were responsible for laboratory study and data analysis. PTS and CR conceived, designed, and performed data analysis as well as wrote and finalized the manuscript. All authors have read, revised, and reviewed the manuscript before submission.

## Funding

This work was supported by the Internal Departmental Funding, Department of Parasitology, and was carried out under Siriraj Integrative Center for Neglected Parasitic Diseases, Faculty of Medicine Siriraj Hospital, Mahidol University. AJ, PR, PTS, and CR also received Chalermphrakiat grants from the Faculty of Medicine Siriraj Hospital, Mahidol University

## Conflict of Interest

The authors declare that the research was conducted in the absence of any commercial or financial relationships that could be construed as a potential conflict of interest.

## Publisher’s Note

All claims expressed in this article are solely those of the authors and do not necessarily represent those of their affiliated organizations, or those of the publisher, the editors and the reviewers. Any product that may be evaluated in this article, or claim that may be made by its manufacturer, is not guaranteed or endorsed by the publisher.
