# Corrigendum: The first molecular genotyping of *Naegleria fowleri* causing primary amebic meningoencephalitis in Thailand with epidemiology and clinical case reviews

**DOI:** 10.3389/fcimb.2022.1021158

**Published:** 2022-09-29

**Authors:** Pannathat Soontrapa, Anupop Jitmuang, Pichet Ruenchit, Supathra Tiewcharoen, Patsharaporn T. Sarasombath, Chatchawan Rattanabannakit

**Affiliations:** ^1^ Division of Neurology, Department of Medicine, Faculty of Medicine Siriraj Hospital, Mahidol University, Bangkok, Thailand; ^2^ Division of Infectious Diseases and Tropical Medicine, Department of Medicine, Faculty of Medicine Siriraj Hospital, Mahidol University, Bangkok, Thailand; ^3^ Department of Parasitology, Faculty of Medicine Siriraj Hospital, Mahidol University, Bangkok, Thailand

**Keywords:** PAM, Primary amebic meningoencephalitis, *Naegleria fowleri*, *Naegleria* spp., free-living ameba, genotyping, ameba

## Text correction

In the published article, there was a typing error in Result statement.

A correction has been made to the Result section, Genotyping and Phylogenetic Analysis Based on ITS1 and 5.8S rRNA Among *N. fowleri* Species Causing PAM in Thailand, Line 11.

The Result statement was previously stated:

“The ITS1 sequences of these cases were compatible with *N. fowleri* genotype T3, which has an ITS1 sequence length of 86 bp and a C nucleotide at position 31 in 5.8S rRNA. The ITS1 nucleotide sequence of the current case No.17 was 100% identical to *N. fowleri* isolate Nf 69 (MZ494674.1). The sequence length of the ITS1 gene in case No.17 was 86 bp, with the T nucleotide at position 31 in the 5.8S rRNA, which indicates genotype T4 as the causative genotype”.

The corrected sentence appears below:

“The ITS1 sequences of these cases were compatible with *N. fowleri* genotype T3, which has an ITS1 sequence length of 86 bp and a T nucleotide at position 31 in 5.8S rRNA. The ITS1 nucleotide sequence of the current case No.17 was 100% identical to *N. fowleri* isolate Nf 69 (MZ494674.1). The sequence length of the ITS1 gene in case No.17 was 86 bp, with the C nucleotide at position 31 in the 5.8S rRNA, which indicates genotype T4 as the causative genotype”.

The authors apologize for this error and state that this does not change the scientific conclusions of the article in any way. The original article has been updated.

## Publisher’s note

All claims expressed in this article are solely those of the authors and do not necessarily represent those of their affiliated organizations, or those of the publisher, the editors and the reviewers. Any product that may be evaluated in this article, or claim that may be made by its manufacturer, is not guaranteed or endorsed by the publisher.

