# Supplementary material for: The First Molecular Genotyping of Naegleria fowleri Causing Primary Amebic Meningoencephalitis in Thailand With Epidemiology and Clinical Case Reviews
Source: Front Cell Infect Microbiol. 2022 Jul 13;12:931546. doi: 10.3389/fcimb.2022.931546 (PMC9326084; doi:10.3389/fcimb.2022.931546)
Supplement: Supplementary file 1 [file Table_1.docx]

**Supplementary Table 1.** Primers and PCR conditions used in this study.

| **Species / target** | **Primers** | **PCR cycling condition** | **Citation** |
| --- | --- | --- | --- |
| Mitochondrial genes | | | |
| 12S rRNA  (693 bp) | 12S Fwd:  5’-GGGTGCGTAGGTTGTGTAATA-3’  12S Rev:  5’-TGGATAGCCCAACCCATTAAG-3’ | 95^◦^C-5 min; 35 cycles of 95^◦^C-30 s, 55^◦^C-30 s, 72^◦^C-1 min; 72^◦^C-5 min, 4^◦^C-forever | Current study |
| 16S rRNA  (836 bp) | 16S Fwd:  5’- AACAAGTACCGTGAGGGAAAG-3’  16S Rev:  5’- CCCTTGGATTTCCGGTGATAA-3’ |  |  |
| Nuclear genes | | | |
| ITS1  (18sRNA, ITS1, 5.8S rRNA, ITS2, and partial 28s rRNA) | ITS1-Fwd:  5′-GAACCTGCGTAGGGATCATTT-3′  Nf ITS1-Rev:  5′-TTTCTTTTCCTCCCCTTATTA-3′ | 94^◦^C-5 min; 35 cycles of 94^◦^C-30 s, 55^◦^C-30 s, 72^◦^C-45 s; 72^◦^C-5 min, 4^◦^C-forever | (Pélandakis et al. , 2000) |
